# Reply to Confalonieri et al. Comment on “Benekos et al. Intraocular Pressure Reduction Following Phacoemulsification in Patients with Exfoliation: A Systematic Review and Meta-Analysis. *J. Clin. Med.* 2024, *13*, 6774”

**DOI:** 10.3390/jcm14082571

**Published:** 2025-04-09

**Authors:** Konstantinos Benekos, Andreas Katsanos, Panagiotis Laspas, Iordanis Vagiakis, Anna-Bettina Haidich, Anastasios G. Konstas

**Affiliations:** 1 Ophthalmology Department, University of Ioannina, 45500 Ioannina, Greece; benekoskonstantinos@gmail.com (K.B.); katsanos@uoi.gr (A.K.); drlaspas@gmail.com (P.L.); 2 1st University Department of Ophthalmology, AHEPA University Hospital Aristotle University of Thessaloniki, Kyriakidi Str., 54636 Thessaloniki, Greece; jvag_@outlook.com; 3 Department of Hygiene, Social-Preventive Medicine and Medical Statistics, School of Medicine, Aristotle University of Thessaloniki, 54124 Thessaloniki, Greece; haidich@auth.gr

We greatly appreciate Confalonieri et al.’s [1] interest in our recently published systematic review and meta-analysis, titled “Intraocular Pressure Reduction Following Phacoemulsification in Patients with Exfoliation: A Systematic Review and Meta-Analysis”. Their comments provide us with the opportunity to further clarify a number of issues regarding the primary focus and methodology of our study and to address the management of intraocular pressure (IOP) using mannitol infusion [2]. To summarize, the key focus of our meta-analysis was to document the effect of cataract surgery on postoperative IOP in patients with either exfoliation syndrome (XFS) or exfoliative glaucoma (XFG). A significant IOP reduction was observed, which may be attributed to the elimination of irido-lenticular friction, which decreases the ensuing release of pigment and exfoliation material from the iris [3]. Further, phacoemulsification surgery washes away exfoliation aggregates and pigment from the outflow system, thus enhancing the aqueous outflow facility and further lowering postoperative IOP in patients with exfoliation [3], while in some cases, deepening the anterior chamber and widening the angle may be contributory.

We agree with Confalonieri et al. that effective IOP control before, during, and after phacoemulsification surgery is essential to protect patients with exfoliation from undesirable IOP spikes that can damage the optic nerve [3]. Unquestionably, prophylactic IOP lowering before surgery could decrease the risk of perioperative and postoperative IOP spikes and wide IOP fluctuations, which are concerning in this at-risk group of patients. The study by Di Maria et al. [4] provides valuable information on the benefits of intravenous mannitol, an osmotic agent that can help significantly in lowering IOP in subjects at risk. However, we should keep in mind that this study is not a randomized controlled trial, which by itself limits the strength of its evidence. In addition, it is worth mentioning that the aim of our meta-analysis was to assess the longer-term effects of phacoemulsification on IOP by evaluating the differences 6 and 12 months post surgery. The study by Di Maria et al. [4] offers valuable insight into the short-term IOP characteristics after the use of mannitol, conducting a single, sitting IOP measurement one hour after surgery. They do not, however, document IOP characteristics beyond this time point, or examine long-term postoperative IOP, which is the focus of our study. Unfortunately, it is difficult to monitor and understand IOP pathology with the use of a single IOP measurement. This distinction remains a drawback in most of the studies we have analyzed and highlights the need for comprehensive documentation of diurnal or 24 h IOP monitoring after surgery and in the long term. Thus, more robust IOP evidence is sorely missing from the literature. Future controlled studies with appropriate diurnal IOP monitoring are desirable to better understand IOP characteristics after phacoemulsification surgery in all subjects, including those with exfoliation. This will optimize long-term IOP management strategies in glaucoma subjects, with an emphasis on XFG patients with cataract who require tight control of IOP fluctuation [3].

In their study, Di Maria et al. [4] administered a fixed dose of 250 mL of 20% mannitol over 30 min in 80 patients, irrespective of their body weight. We would suggest that it may be advisable to individualize the exact dosing and duration of mannitol administration by calculating the suitable dosing of mannitol per kilo of body weight. Future research is needed to determine the optimal individualized mannitol dosage aimed at obtaining maximum IOP reduction whilst minimizing the potential side effects. Another effective alternative, with potentially fewer side effects, is the preoperative and postoperative use of oral acetazolamide [5,6], which also reduces IOP spikes during and after phacoemulsification surgery. This is the preferred option for at-risk exfoliation patients in our practice. Direct head-to-head comparative studies on exfoliation patients receiving either intravenous mannitol or oral acetazolamide need to be performed to further elucidate their relative efficacies and safety.

Di Maria et al. [4] suggested that preoperative intravenous mannitol significantly reduces IOP in both phakic and pseudophakic eyes, irrespective of changes in anterior chamber depth or axial length. However, as their study assessed the contralateral eyes rather than the eye undergoing surgery, it is unclear how their findings relate to the outcome of preoperative IOP optimization in at-risk exfoliation eyes.

We should emphasize that the studies we included in our meta-analysis primarily assessed uncomplicated phacoemulsification cases. It is well documented that cataract surgery in patients with XFS and particularly XFG poses significant challenges, due to a higher incidence of intraoperative complications. These complications are primarily related to insufficient pupillary dilatation and a higher prevalence of zonular instability, which increase the rate of posterior capsular breaks and vitreous loss [3]. Additional factors contributing to the increased surgical risk include a shallower anterior chamber, corneal endothelial alterations, and the disruption of the blood–aqueous barrier [3]. It is not clear to what extent surgical complications in exfoliation eyes can negatively impact IOP control. The primary focus of our study was to calculate the net effect on IOP of phacoemulsification under controlled conditions. It is conceivable, however, that studies including cases with intraoperative complications might have influenced the IOP results, or could have increased the heterogeneity between studies.

Finally, we agree with the comment by Confalonieri et al. concerning the low certainty of evidence in our meta-analysis. We emphasize the need for more methodologically rigorous research on this topic. Key limitations include the lack of consideration for the eye-to-eye correlation and the inclusion of data from both eyes of some patients, without applying the appropriate statistical adjustments. The absence of washout periods before the IOP measurements or the absence of multiple IOP measurements at both baseline and follow-up visits clearly introduce bias in all the studies we have analyzed (that evaluate IOP changes before, during, and after surgery). Addressing these issues in the future will be essential in improving the reliability of the available evidence. This will facilitate the development of evidence-based guidelines for successful IOP management in patients with exfoliation undergoing phacoemulsification surgery.

## References

[1] Confalonieri F., Ferraro V., Barone G., Gaeta A., Di Maria A. (2025). Comment on Benekos et al. Intraocular Pressure Reduction Following Phacoemulsification in Patients with Exfoliation: A Systematic Review and Meta-Analysis. *J. Clin. Med.* 2024, *13*, 6774. J. Clin. Med..

[2] Benekos K., Katsanos A., Laspas P., Vagiakis I., Haidich A.-B., Konstas A.G. (2024). Intraocular pressure reduction following phacoemulsification in patients with exfoliation: A systematic review and meta-analysis. J. Clin. Med..

[3] Konstas A.G., Mikropoulos D.G., Teus M.A., Katsanos A., Aristeidou A., Voudouragkaki I.C., Kozobolis V.P., Holló G. (2025). Cataract surgery in patients with exfoliation syndrome. Cataract Surgery in the Glaucoma Patient.

[4] Di Maria A., Ferraro V., Barone G., Gaeta A., Vinciguerra P., Confalonieri F. (2025). Preoperative intravenous mannitol administration and its rationale before cataract surgery. Graefe’s Arch. Clin. Exp. Ophthalmol..

[5] Hayashi K., Yoshida M., Sato T., Manabe S., Yoshimura K. (2018). Intraocular pressure elevation after cataract surgery and its prevention by oral acetazolamide in eyes with pseudoexfoliation syndrome. J. Cataract. Refract. Surg..

[6] Hayashi K., Yoshida M., Manabe S., Yoshimura K. (2017). Prophylactic effect of oral acetazolamide against intraocular pressure elevation after cataract Surgery in eyes with glaucoma. Ophthalmology.

